# Rat thalamic neurons encode complex combinations of heading and movement directions and the trajectory route during translocation with sensory conflict

**DOI:** 10.3389/fnbeh.2014.00242

**Published:** 2014-07-21

**Authors:** Nyamdavaa Enkhjargal, Jumpei Matsumoto, Choijiljav Chinzorig, Alain Berthoz, Taketoshi Ono, Hisao Nishijo

**Affiliations:** ^1^System Emotional Science, Graduate School of Medicine and Pharmaceutical Sciences, University of ToyamaToyama, Japan; ^2^Center for Interdisciplinary Research in Biology, Collège de FranceParis, France

**Keywords:** thalamus, head direction, sensory conflict, vestibular, optic flow

## Abstract

It is unknown how thalamic head direction neurons extract meaningful information from multiple conflicting sensory information sources when animals run under conditions of sensory mismatch. In the present study, rats were placed on a treadmill on a stage that moved in a figure-8-shaped pathway. The anterodorsal and laterodorsal neurons were recorded under two conditions: (1) control sessions, in which both the stage and the treadmill moved forward, or (2) backward (mismatch) sessions, in which the stage was moved backward while the rats ran forward on the treadmill. Of the 222 thalamic neurons recorded, 55 showed differential responses to the directions to window (south) and door (north) sides, along which the animals were translocated in the long axis of the trajectory. Of these 55 direction-related neurons, 15 showed heading direction-dependent responses regardless of movement direction (forward or backward movements). Thirteen neurons displayed heading and movement direction-dependent responses, and, of these 13, activity of 6 neurons increased during forward movement to the window or door side, while activity of the remaining 7 neurons increased during backward movement to the window or door side. Eighteen neurons showed movement direction-related responses regardless of heading direction. Furthermore, activity of some direction-related neurons increased only in a specific trajectory. These results suggested that the activity of these neurons reflects complex combinations of facing direction (landmarks), movement direction (optic flow/vestibular information), motor/proprioceptive information, and the trajectory of the movement.

## Introduction

Head direction (HD) cells, which are neurons that fire when the animal is facing a particular direction relative to a fixed location or landmark in the environment, are believed to represent the animal's perceived directional heading in its environment (Taube et al., 1990a,b). Recent studies have reported that information from HD cells is processed in place and grid cells to form a spatial representation (cognitive map) of the environment (O'Keefe and Nadel, 1978; Moser and Moser, 2008), and it is critical for accurate navigation in situations that require a flexible allocentric cognitive mapping strategy (Gibson et al., 2013). It has been reported that up to 10 different brain structures contain neurons selective for HD, including the anterodorsal (AD) and laterodorsal (LD) thalamic nuclei (Taube, 2007). The LD and AD nuclei receive visual inputs from the retrosplenial cortex (RSC) and the postsubiculum, which are reciprocally connected with both the AD and LD thalamic nuclei (Vogt and Miller, 1983; Sripanidkulcha and Wyss, 1986), and vestibular and motor information from the lateral mammillary nucleus (Taube, 2007). It is reported that AD and LD neurons showed head direction-selective responses, which extinguished in darkness (Mizumori and Williams, 1993; Taube, 1995).

The exact mechanism by which the HD signal is generated remains unknown, but it is clearly dependent upon multiple sensory modalities, including vestibular, visual, and proprioceptive inputs (Goodridge and Taube, 1995; Taube et al., 1996; Stackman and Taube, 1997; Stackman et al., 2003; Muir et al., 2009). These multiple sensory cues are integrated to reduce HD errors (Telford et al., 1995; Becker et al., 2002; Fetsch et al., 2010; Frissen et al., 2011). Recent studies have investigated the effects of sensory conflict (mismatch) among these sensory inputs to the HD system (Chen et al., 1994; Goodridge and Taube, 1995; Blair and Sharp, 1996; Knierim et al., 1998; Zugaro et al., 2001a,b, 2002; Stackman et al., 2003; Yoder et al., 2011). When visual (landmarks) cues conflict with vestibular/proprioceptive ideothetic cues, AD thalamic HD cells have been reported to follow visual or vestibular/proprioceptive cues, depending on the mismatch magnitudes (Knierim et al., 1998). These previous results suggest that the HD system might function differently in ordinary and conflicting conditions; in a conflicting situation, instead of integrating the multiple sensory inputs to reduce the error of the heading direction, the HD system might extract meaningful information among the multiple sensory inputs.

In our previous studies, rats were placed on a treadmill affixed to a moving stage and the rats were moved backward by translocation of the stage while the rats ran forward on the treadmill. In this backward translocation condition, idiothetic sensory inputs [visual (optic flow), vestibular inputs, and proprioceptive inputs or motor efferent copy] were mismatched; the proprioceptive inputs and/or motor efferent copy during locomotion (locomotion-related inputs) did not match the visual (optic flow)/vestibular inputs. Notably, although the rats showed increases in hippocampal theta power and sympathetic nervous activity, which is similar to symptoms in motion sickness (Zou et al., 2009a; Aitake et al., 2011), these changes returned to the baseline level after repeated exposure to the conflicting situation (Zou et al., 2009a; Aitake et al., 2011). These results suggest that the animals could adapt to this conflicting situation after repeated training, and that the rats could normally recognize the space in this condition. It is noted that, during the backward translocation, information of head direction cannot predict the destination where the rat is reaching, since updating the current location requires information of movement direction instead of head direction. This suggests that the HD system might extract movement direction signals from the multiple sensory inputs. It is recently reported that entorhinal HD cells represent only the head direction but not the movement direction (Cei et al., 2014). Therefore, HD cells in the other brain regions might take such a role. In the present study, we investigated the direction-related responses of AD and LD thalamic neurons during the backward translocation.

The second purpose of the present study is to investigate effects of trajectory routes on the HD cell activity. A human behavioral study reported that head and eyes systematically deviated toward the future direction of the curved trajectory (Grasso et al., 1998). This kind of anticipatory orientation would allow for achieving a stable reference frame in time to effectively program and execute action (Grasso et al., 1998). Consistently, directional tuning of thalamic HD cells in the rat systematically displays anticipatory shifts toward the future direction of the head in space (Blair and Sharp, 1995). These results suggest that HD cells calculate the current directional heading by combining information about the previous head direction and the velocity at which the head is turning (McNaughton et al., 1991). On the other hand, hippocampal place cells were reported to differently respond to a place depending on the routes (Wood et al., 2000; Dayawansa et al., 2006). In the same way, we hypothesized that HD cell activity would be influenced by trajectory routes. To investigate this issue, we recorded the neural activity during the rats along a figure-8-shaped track, navigated by two different routes that shared a common central stem.

## Materials and methods

### Subjects

Twenty-five Wistar male rats weighing 200–300 g were used. They were individually housed in cages controlled at a constant temperature (20 ± 1°C) with free access to water and laboratory chow. After 1 week of acclimatization, they underwent an operation to implant a head cap on the skull. All rats were treated in strict compliance with the United States Public Health Service Policy on Human Care and Use of Laboratory Animals, the National Institutes of Health Guide for the Care and Use of Laboratory Animals, and the Guidelines for the Care and Use of Laboratory Animals at the University of Toyama. The present study has been approved by the Ethical Committee of Animal Experiments in University of Toyama.

### Surgery

The rats were anesthetized with pentobarbital sodium (40 mg/kg, intraperitoneal) and then fixed in a stereotaxic apparatus. A cranioplastic cap was attached to the skull, as described in our previous studies (Nishijo and Norgren, 1990; Uwano et al., 1995). After the surgery, an antibiotic (gentamicine sulfate) was administered topically and systemically (2 mg, intramuscular). The main function of this cranioplastic cap is to provide artificial ear bars, which were used in the subsequent experiment, because the cranioplastic cap can be painlessly fixed into the stereotaxic apparatus while the rats are running on the treadmill. After 1 week of recovery, the rats were trained for 2–3 weeks in the forward condition.

After 2–3 weeks of training (see Training), the rats were reanesthetized, and the cranioplastics on the heads were fixed by the ear bars into the stereotaxic apparatus on the mobile stage. A hole (3–5 mm diameter) for the semichronic recordings was drilled through the cranioplastic cap and the underlying skull (−1.8 to −3.6 mm anterior and 1.0–3.0 mm lateral from the bregma) according to the atlas of Paxinos and Watson (1998). The exposed dura was excised, and the hole was covered with hydrocortisone ointment. The hole was covered with a sterile Teflon sheet and sealed with epoxy glue.

### Apparatus and tasks

**Figure 1:**
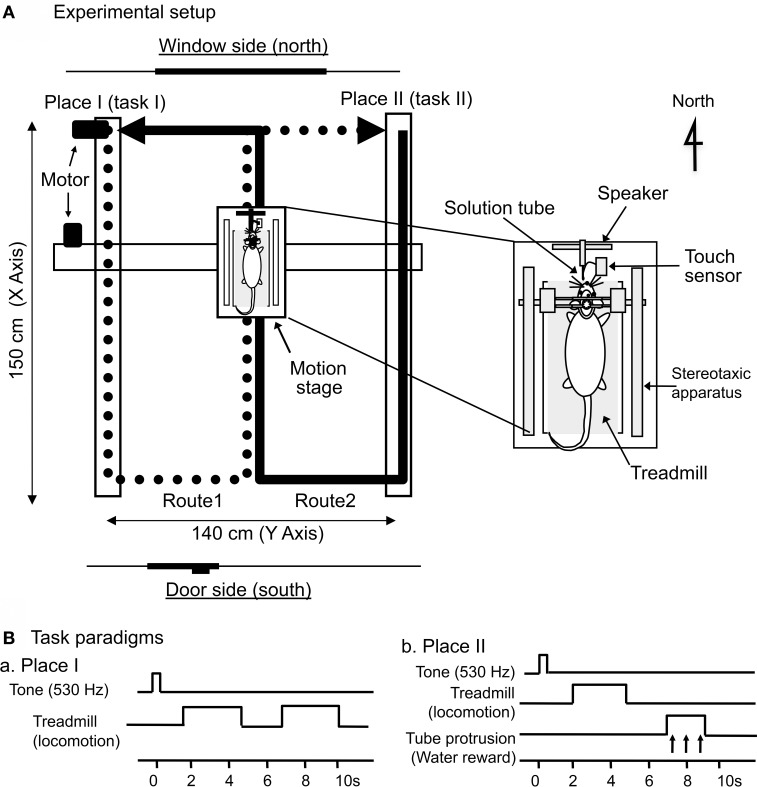
**Schematic illustration of the experimental setup. (A)** Mobile stage translocation device. A computer-controlled device carried the mobile stage on a figure-8-shaped route, and the start point of each route was designated as Place I or Place II, respectively. The arrows indicate the movement direction of the mobile stage. The inset indicates the mobile stage. A stereotaxic apparatus and a treadmill were attached to the mobile stage, and the rat was placed on the treadmill inside a spacious transparent plastic enclosure. **(B)** Paradigms of the delayed stimulus-response association (DSR) task that was conducted at Places I and II. At Place I (Ba), the task was initiated by a cue tone followed by 2 periods of 3.0 s, during which the treadmill rotated. At Place II (Bb), the task was initiated by the same cue tone followed by a 3.0-s period of treadmill rotation and a 2.0-s period of tube protrusion. The tone and the following treadmill operation were separated by 1.5-s intervals, while the 2 reinforcements were separated by 2.0-s intervals.

The same apparatus as that in our previous papers (Dayawansa et al., 2006; Zou et al., 2009a,b; Aitake et al., 2011) was used. A stereotaxic apparatus with a transparent plastic enclosure for the semichronic recording (Nishijo and Norgren, 1990) and a treadmill were attached to the mobile stage (Figure 1A). The floor of the enclosure was removed so that the rats could run on the treadmill. The rats' heads were painlessly fixed to a stereotaxic frame on the mobile stage, which was driven horizontally by belts that had two motors affixed to the horizontal frames of the base for X-Y coordinates; another motor, which was attached to the base, rotated the rat so that it faced in the direction of translocation (THK Co., Ltd., Kanazawa, Japan). The mobile stage moved between Places I and II at a speed of 20 cm/s in a figure-8-shaped pathway that consisted of Routes 1 and 2. The treadmill was driven at the same speed (20 cm/s) as the translocation of the stage. Route 1 connected Place I and Place II along the trace indicated by the thick solid line in Figure 1A, and Route 2 connected Place I and Place II along the trace indicated by the thick dotted line in Figure 1A. Thus, Routes 1 and 2 shared a common central stem in the figure-8-shaped pathway. At the corners of the figure-8-shaped pathway, except for at Places I and II, the trajectory of the mobile stage was smoothed with a clothoid curve (i.e., curvature is equal to its arc length) with minimum radius of curvature 12 cm during angular rotation. The total length of each route was 440 cm, and the central stem was 150 cm in length.

**Figure 2:**
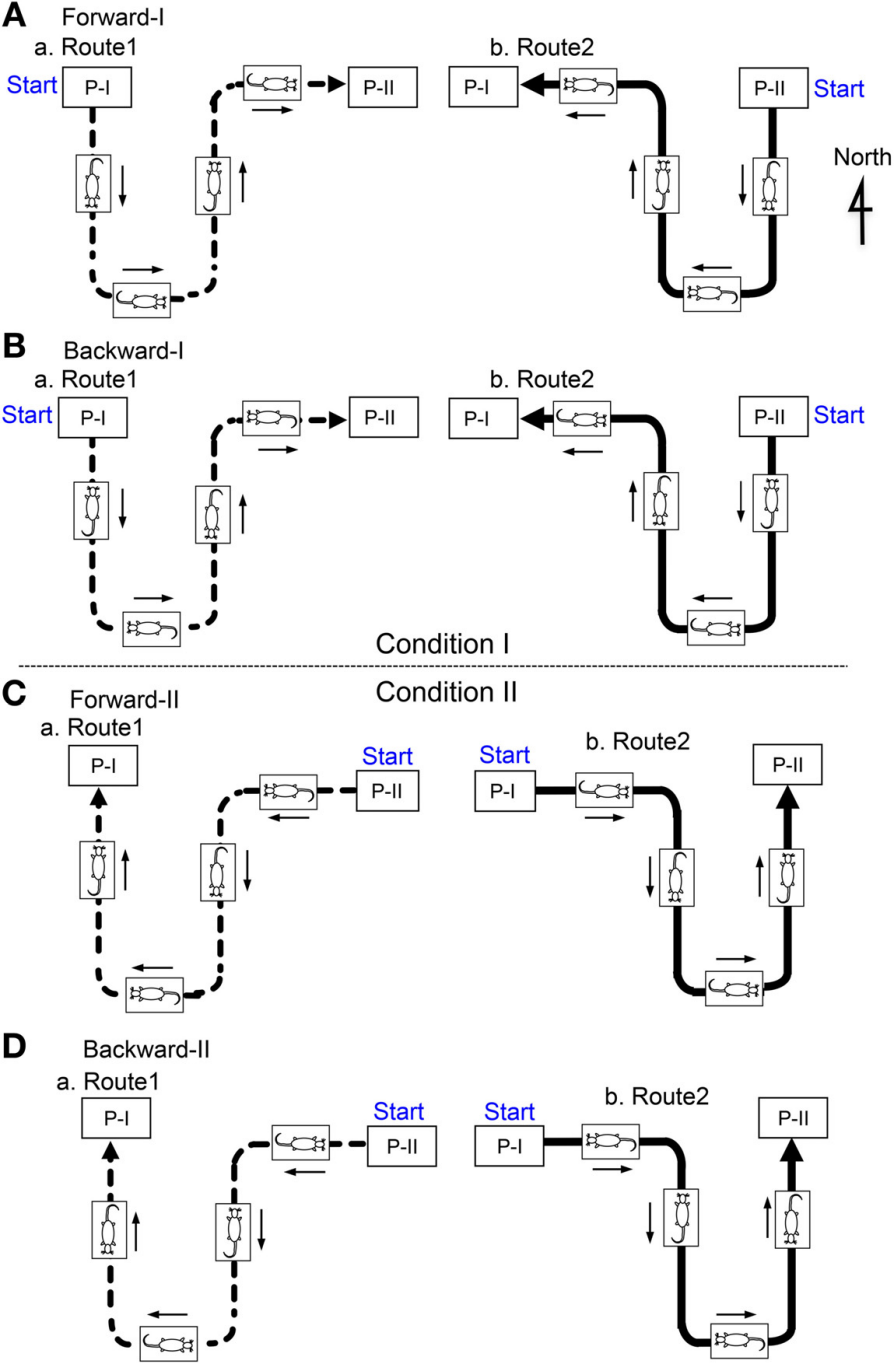
**The position of the mobile stage and the animal as well as the direction of the rat's movement in several consecutive phases of the passage in the figure-8 pathway in the Condition I (A, Forward-I; B, Backward-I) and II (C, Forward-II; D, Backward-II).** In the Condition II (Forward-II and Backward-II), the position of the mobile stage was similar, but the movement directions of the mobile stage were opposite to those in the Condition I (Forward-I and Backward-I). P-I, Place I; P-II, Place II.

There were four translocation tasks, and Figure 2 illustrates the position of the mobile stage and of the animal, as well as the direction of the rats' movements, in several consecutive phases of the passage through the bent parts of Routes 1 (a) and 2 (b) in each task. In the forward-I task (A), the mobile stage started from Place I and ended at Place II (Aa) on Route 1, and, on Route 2, the stage started from Place II and ended at Place I (Ab). The rats always faced toward the direction of the tangent of the translocation routes in order to imitate the changes in direction in natural locomotion. At Places I and II, the mobile stage stopped, and tasks were imposed on the rats (see below in detail). After the task, the mobile stage was rotated before translocation so that the rat faced in the direction of translocation in Routes 1 and 2. In the second (backward-I) task (B), the mobile stage was turned around by 180° before the translocation and then similarly translocated. In this task, although the rat ran forward on the treadmill, the mobile stage was translocated backward in terms of the rats' direction. In the third and fourth (forward-II and backward-II) tasks (C,D), the movement direction of the mobile stage was opposite to those in the first (forward-I) and second (backward-II) tasks, respectively.

In each translocation task, the stage paused at the two corners of the pathway that were designated Places I and II, and this was where the rats performed a delayed stimulus-response association (DSR) task (see later). These two stops on the way divided the figure 8-shaped track into two routes (Routes 1 and 2) with a common central stem.

To associate specific places on the track with positive (rewarding) and negative (non-rewarding) episodes, a distinctive DSR task with no reward and with reward were conducted at Places I and II, respectively (Figure 1B). A speaker located anterior to the rats' heads delivered a pure tone (530 Hz) for 0.5 s. A small tube was automatically protruded close to the rats' mouths for 2.0 s in order to evoke water licking, which was signaled by a touch sensor triggered when the tongue made contact with the tube. At Place I (Ba), the DSR task was initiated by the tone for 0.5 s. After a 1.5-s delay, the treadmill was operated at 20 cm/s for 3.0 s; after a 2.0-s interval in which the treadmill was stopped, it was operated again at the same speed for another 3.0 s. Each of these 3.0-s runs reliably induced locomotion without reward. At Place II (Bb), the task was initiated by the same tone, and the treadmill began to move (at the same speed and for the same duration as above) after a 1.5-s delay. The tube was then protruded in front of the rats' mouths for 2.0 s. If the rat licked the tube during this period, it could ingest a water reward.

### Training

The rats were trained to adapt to the recording environment step by step as described below. The rats were acclimatized by handling for several days, and familiarized to being placed for short periods in the plastic restraining enclosure used for the task, but not fixed to the stereotaxic frame. This initial adaptation procedure was executed for 10–20 min/day, and the period for which the rats were placed in the enclosure was gradually increased. This step was repeated before and after the surgery. Then, their heads were fixed to the stereotaxic frame, and the treadmill was driven. Speed of the treadmill gradually increased day by day. Then, they were trained to adapt to the enclosure while the stage was moving on the track. Finally we trained them to perform the DSR tasks. Under a 22 h water-deprivation regimen, the rats were trained to carry out the DSR task at Places I and II and to run on the treadmill while the motion stage was translocated. Throughout the training and the subsequent recording period, the rats were permitted to ingest 20–30 ml of water while in the restrainer. If a rat failed to drink a total volume of 30 ml water while restrained, it was given the remainder when it returned to its home cage. After these procedures, the rats accepted the restraint condition in the enclosure without struggling. After training in the forward condition for 2 weeks, and forward and backward condition for 5 days, the rats were subjected to the experimental sessions.

### Recording

Each rat was usually tested every other day. After the rat was placed in the enclosure, the sterile Teflon sheet was removed, and a tritrode (a 4-cores-Quarts-Platinum/Multifiber electrode, Thomas RECORDING GmbH, Giessen, Germany) (*Z* = 0.5–1.5 MΩ at 1 kHz) was stereotaxically inserted stepwise with a pulse motor-driven manipulator (SM-20, Narishige Scientific Instrument Lab, Tokyo, Japan) into the AD and LD thalamic nuclei. The analog signals of the neuronal activities, the triggers for the tone, the water reward, the licking, and the X-Y coordinates of the mobile stage were digitized and stored in a computer through a Multichannel Acquisition Processor (MAP, Plexon Inc., Dallas TX) system.

The digitized neuronal activities were isolated into single units by their waveform components with the Offline Sorter program (Plexon Inc.). The waveforms of the isolated units were superimposed in order to check for invariability throughout the recording sessions, and they then were transferred to the NeuroExplorer program (Nex Technologies, Madison, AL, USA) for further analysis. When the neurons were isolated, their activities were recorded while the rats performed the forward and backward tasks and the DSR task. Every task always started at Place I in Route 1 in the forward-I and backward-I tasks (Condition I) and at Place I in Route 2 in the forward-II and backward-II tasks (Condition II). Each translocation task consisted of 3 laps of translocation. During each lap (Routes 1 and 2), 3 trials of the DSR task were given at Places I and II, respectively.

In 18 rats, the neurons were initially tested in the forward-I task and then tested in the backward-I task (Condition I). When the neuronal activity was still located, the same recording was repeated in these two movements. In four rats, the neurons were initially tested in the forward-II task and then tested in the backward-II task (Condition II). In three rats, the neurons were tested in both the Condition I and II.

### Data analysis

The neurons were tested with at least both the forward and backward tasks. Each route was divided into 56 successive pixels, and the firing rate maps of Routes 1 and 2 were separately constructed. First, the mean firing rate for each pixel was calculated as the average number of spikes per second for all visits to that pixel during translocation. Then, the firing rate maps were reconstructed with a smoothing method. The smoothed firing rate of a given pixel was defined as the mean of 3 pixels (the given pixel and the 2 adjoining pixels). This firing rate map was separately created in Routes 1 and 2 in the individual tasks, including the forward-I, backward-I, forward-II, and backward-II tasks.

The direction at which a given neuron fired maximally was defined as the neuron's preferred firing direction for the heading direction and/or movement direction related responses. In the present study, only the neurons that displayed a preferred direction to the window (north) or door (south) side were analyzed (Figure 1A). The direction-related neurons were defined in each route in each translocation task as follows: (1) the maximal firing rate at its preferred firing direction (maxPFR) should be greater than both the maximal firing rate at the opposite direction (maxOPFR) and the average firing rate during the linear movement to the window (north) and door (south) sides in each route; (2) the maxPFR should be greater than 2 times the average firing rate in the whole pathway in each route; and (3) the selectivity index (SI) for the preferred firing direction should be greater than 1.0. The SI was defined by the following formula:

SI = (maxPFR – maxOPFR)/Mean firing rate during whole translocation (Routes 1 and 2) across the translocation conditions.

Statistical significance of the direction-related neurons selected by the above criteria was tested and further classified using the following ANOVAs. First, the average firing rates of the pixels in each stem along the north-south axis were compared by Three-Way ANOVA with heading direction (north and south), movement direction (north and south), and route (Routes 1 and 2) as factors. The head direction-related neurons were defined as the neurons with a significant main effect of heading direction (*p* < 0.05) and without the significant main effect of moving direction (*p* > 0.05). The movement direction-related neurons were defined as neurons with a significant main effect of movement direction (*p* < 0.05) and without a significant main effect of heading direction (*p* > 0.05). The neurons without significant main effects of heading and movement directions and without a significant interaction between heading and movement directions were considered to be non-responsive neurons.

Second, the remaining neurons (i.e., neurons with significant main effects of both head and movement directions, and those with a significant interaction between head and movement directions) were further analyzed by Two-Way ANOVA with heading direction and route as factors in each of the forward and backward tasks. The forward movement-related neurons were defined as neurons that showed a significant main effect of heading direction in the forward task but not in the backward task. The backward movement-related neurons were defined as neurons that showed a significant main effect of heading direction in the backward task but not in the forward task. The remaining neurons were classified as the miscellaneous direction-related neurons. Significant modulation by the routes was defined as such if the neurons showed a significant interaction between direction and route.

The activities of the thalamic neurons during the DSR task were analyzed by creating perievent histograms aligned with the tone onset in the task separately at Places I and II. However, no significant changes in activity were observed in the DSR (data not shown).

### Histology

Upon completion of all the experiments, each rat was reanesthetized with sodium pentobarbital (50 mg/kg, i.p.) and several small electrolytic lesions (80 μA for 60 s) were made stereotaxically around the recorded sites with a glass-insulated tungsten microelectrode. Then, the rats were perfused intracardially with saline and 4% formaldehyde. The brains were extracted and stored in formaldehyde, and frozen sections (30 μm) were cut coronally, stained with cresyl violet. All marking and stimulation sites were then carefully verified microscopically, and shrinkage of the brain was computed based on the distance between the marking lesions on the tissue sections. Positions of neurons were sterotaxically located on the real tissue sections in each animal since stereotaxic coordinates of all marking lesions and recording sites were determined in reference to the same reference pins embedded in the cranioplastic acrylic. Finally the recording sites were re-plotted on the corresponding sections on the atlas of Paxinos and Watson (1998).

The averaged recording positions along the anterior-posterior axis of each type direction-related neurons in the AD and LD were compared using One-Way ANOVA and following *post-hoc* pairwise comparisons (Ryan's method; *p* < 0.05).

## Results

**Figure 3:**
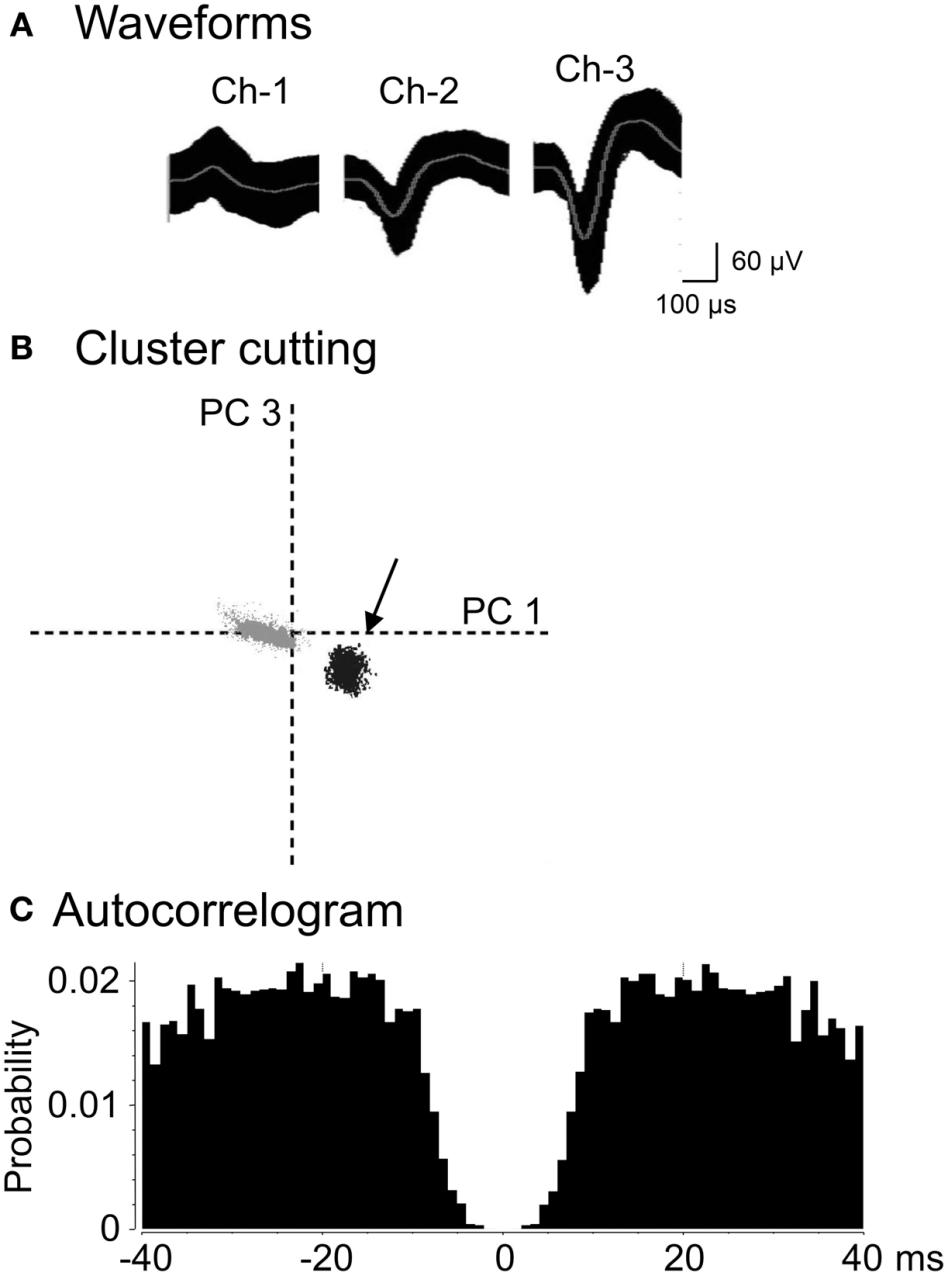
**An example of the raw records of a thalamic neuron. (A)** Superimposed waveforms recorded from 3 electrodes (TriTrode). Chs. 1–3 indicate the signals from individual electrodes. **(B)** The results of the off-line cluster analysis. Each dot represents 1 neuronal spike. Only 1 cluster (indicated by an arrow) was recognized. The horizontal axis represents the principle component 1 (PC1), and the vertical axis represents the principle component 3 (PC3). **(C)** Autocorrelograms of the neurons indicated in **(A,B)**. Bin width, 1 ms. The ordinates indicate probability and the number of spikes per bin.

A total of 222 neurons were recorded from the AD and LD thalamic nuclei of 25 rats. Figure 3 shows an example of the raw records of a thalamic neuron. The typical waveforms, which were simultaneously recorded from the same tritrode (Chs. 1–3), of 1 thalamic neuron are shown in Figure 3A. In contrast to the rat hippocampus, usually one, and only occasionally 2–3 neurons per tritrode, were encountered in the rat thalamus. Figure 3B shows the results of spike sorting by the off-line cluster cutting of the neural activity shown in Figure 3A. Each dot represents 1 spike, and the cluster of dots encircled by dotted lines was easily recognized. Figure 3C shows an autocorrelogram of the neuron shown in Figure 3B. The autocorrelogram indicated that the refractory period of the neuron was 2–3 ms, which indicated that these spikes were recorded from a single neuron.

### Classification of the direction-related neurons

The direction-related cells were defined in each route in each task. Of the 222 neurons, 55 neurons showed direction-related responses (Table 1; see Materials and Methods for the details of the classification). Of the 55 direction-related neurons, 15 (15/55, 27.3%) neurons showed heading direction-dependent responses regardless of movement direction. Thirteen (13/55, 23.6%) neurons displayed direction-related responses that were dependent on both heading and movement direction. Of these 13, the activity of 6 neurons increased in the forward tasks (forward movement-related neurons), while the activity of the 7 neurons increased in the backward tasks (backward movement-related neurons). Eighteen (18/55, 32.7%) neurons showed movement direction-related responses regardless of facing direction.

**Table 1:** **Classification of Anterodorsal (AD) and Laterodorsal (LD) thalamic neurons**.

	**No. of neurons**			**Route-modulation (+)**			**Route-modulation (-)**		
	**AD**	**LD**	**Total**	**AD**	**LD**	**Total**	**AD**	**LD**	**Total**
Head direction-related	4	11	15	3	7	10	1	4	5
Forward movement-related	2	4	6	1	2	3	1	2	3
Backward movement-related	1	6	7	0	2	2	1	4	5
Movement direction-related	8	10	18	6	1	7	2	9	11
Miscellaneous direction-related	3	6	9						
Subtotal	18	37	55						
No responses	46	121	167						
Total	64	158	222						

Route-modulation (+), (−), significant and non-significant interaction between “direction” and “route” in ANOVA, respectively.

### Heading direction-related neurons

**Figure 4:**
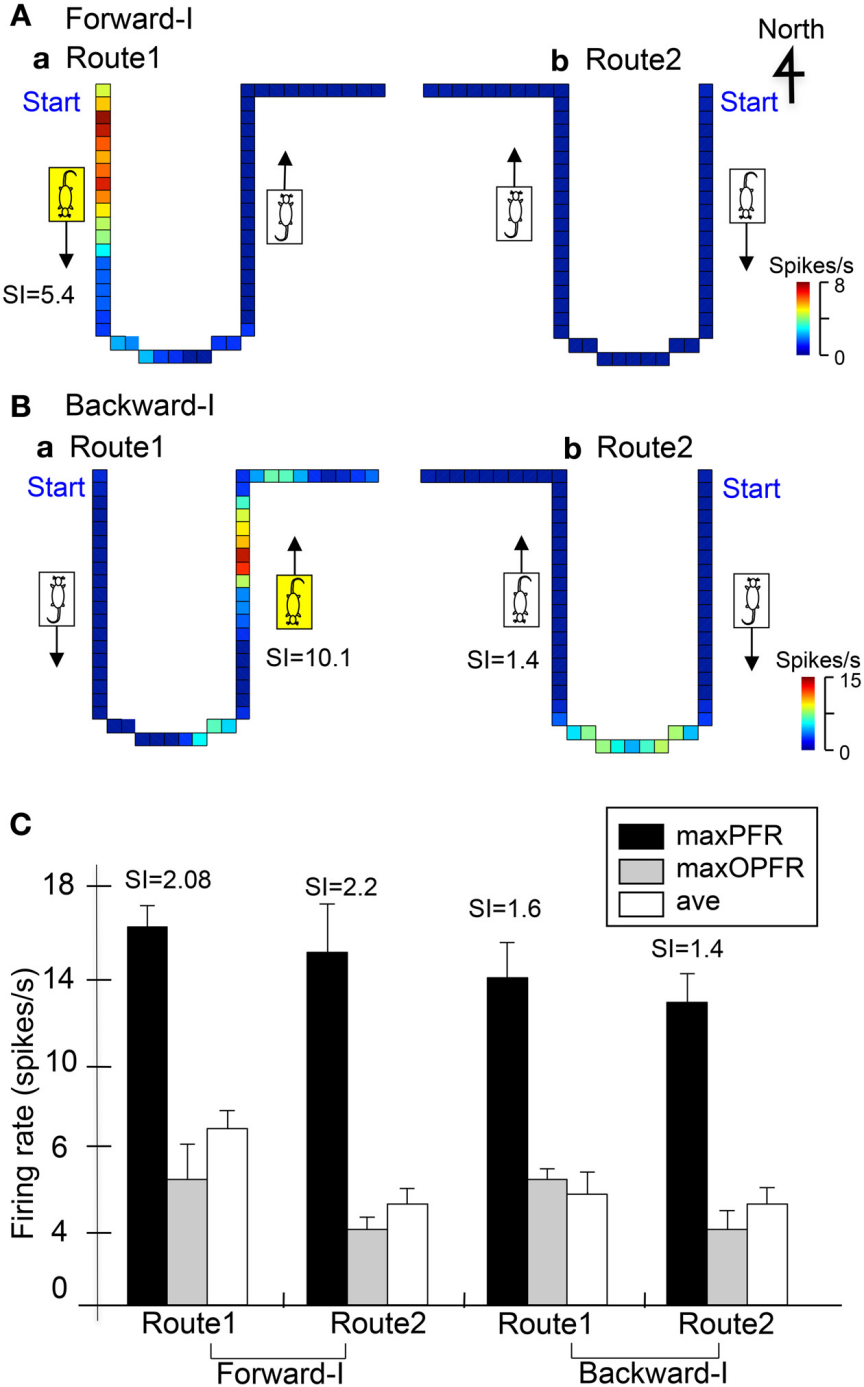
**Two examples of heading direction-related neurons in LD. (A,B)** Firing rate maps of the same neuron in Routes 1 and 2 in the Forward-I **(A)** and Backward-I **(B)** tasks. In the Forward-I task **(A)**, the activity of the neuron increased when the stage moved to the door side (south) only in Route 1 but not when the stage moved to the window side (north) in both Routes 1 and 2. In the Backward-I task **(B)**, the activity of the neuron increased when the stage moved to the window side (north) only in Route 1 but not when the stage moved to the door side (south) in Routes 1 and 2. Thus, the activity increased when the rat faced the south in Route 1. The color of each pixel indicates the neuronal activity calibrated at the right bottom (spikes/s). The arrows indicate the movement direction of the mobile stage. **(C)** Response summary of another heading direction-related neuron. Ordinate indicates firing rate of the neuron. Black, gray, and white columns indicate maximal firing rates in the preferred direction (maxPFR), maximal firing rates in the opposite direction (maxOPFR), and averaged firing rates in the route (ave), respectively. Error bars indicate s.e.m.

Figures 4A,B shows representative data of a heading direction-related neuron in LD tested in the Condition I. In the forward-I task, the activity of the neuron increased in Route 1 when the rat faced the door side (south) during forward movement (A). The SI index of this activity increase was 5.4. In the backward-I movement, the activity of the neuron increased in Route 1 when the rat faced the door side (south) during backward movement (B). The SI index of this activity increase was 10.1. Thus, the activity of the neuron increased when the rat faced the door side (south) in Route 1 regardless of movement direction.

### Forward movement-related neurons

**Figure 5:**
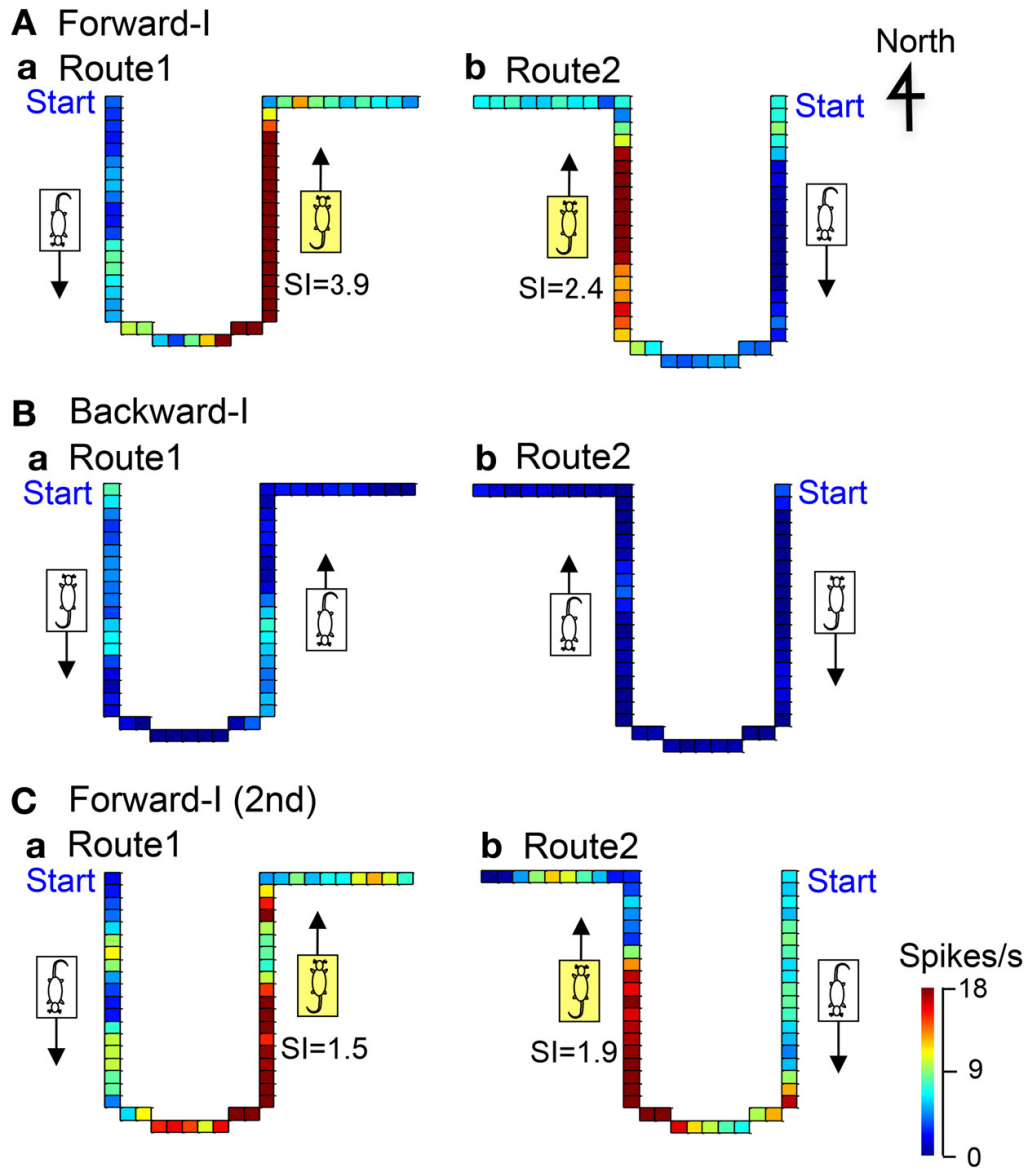
**An example of a forward movement-related neuron in LD.** In the Forward-I task **(A,C)**, the activity of the neuron increased when the stage moved to the window side (north) in both Routes 1 and 2 but not when the stage moved to the south. In the Backward-I task **(B)**, no differential activity changes were observed. Thus, the activity increased during forward movement to the window side (north). The descriptions are the same as for Figure 4.

Figure 5 shows representative data of a forward movement-related neuron in LD tested in the Condition I. In the forward-I task, the activity of the neuron increased when the stage moved to the window side (north) in both Route 1 (*SI* = 3.9) and Route 2 (*SI* = 2.4), but it did not increase when the stage moved to the door side (south) in both Routes 1 and 2 (A). In the backward-I task, no activity changes were observed in both Routes 1 and 2 (B). Furthermore, this neuron was tested again in the forward-I task, and it displayed similar forward movement-related responses (C). Thus, the activity of this neuron increased during forward movement to the window side (north).

### Backward movement-related neurons

**Figure 6:**
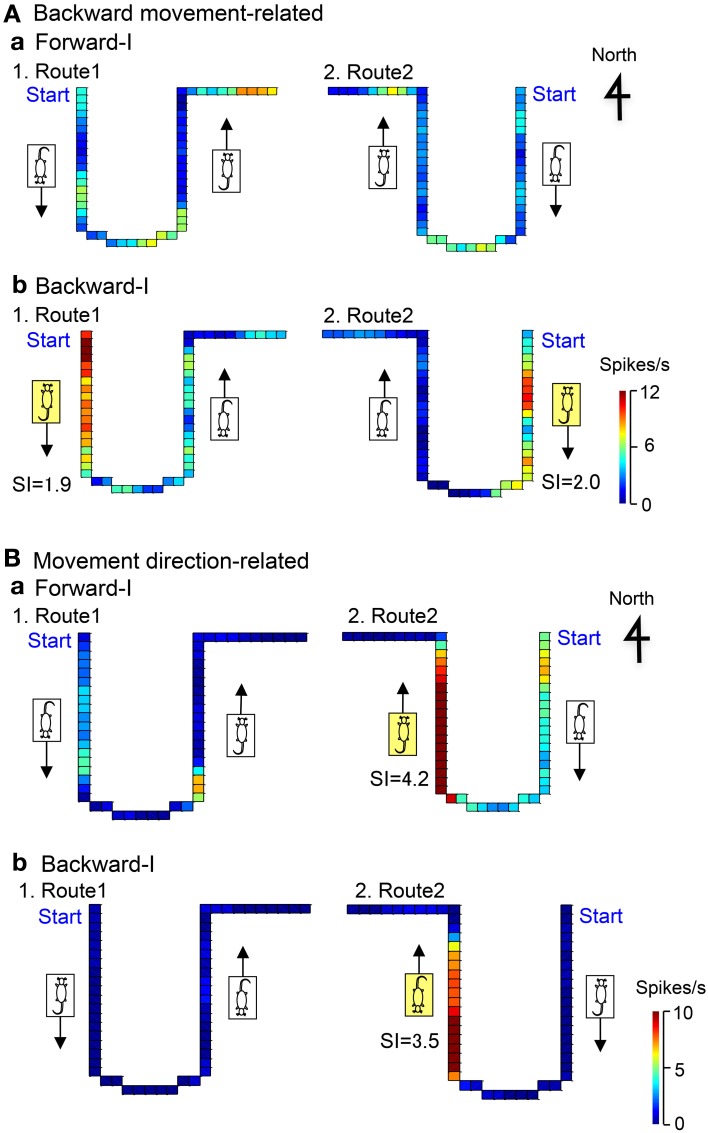
**Examples of backward movement-related neuron in LD (A), and movement direction-related neuron in AD regardless of heading direction (B). (A)** In the Forward-I task (a), no direction-related responses were observed. In the Backward-I task (b), the activity of the neuron increased when the stage moved to the door side (south) but not when the stage moved to the window side (north). Thus, the activity increased during backward movement. The descriptions are the same as for Figure 4. **(B)** In the Forward-I task (a), the activity of the neuron increased when the stage moved to the window side (north) only in Route 2 but not when the stage moved to the south in both Routes 1 and 2. In the Backward-I task **(B)**, the activity of the neuron similarly increased when the stage moved to the window side (north) in Route 2. Thus, the activity increased when the stage moved to the north regardless of the f heading direction of the rat. The descriptions are the same as for Figure 4.

Figure 6A illustrates the representative data of a backward movement-related neuron in LD tested in the Condition I. In the forward-I task, no direction-related responses were observed in both Routes 1 and 2 (a). In the backward-I task, the activity of the neuron increased when the stage moved to the door side (south) in both Route 1 (*SI* = 1.9) and Route 2 (*SI* = 2.0), but it did not increase when the stage moved to the window side (north) in both Routes 1 and 2 (b). Thus, the activity of this neuron increased during backward movement to the door side (south).

### Movement direction-related neurons

Figure 6B illustrates the representative data of a movement-related neuron in AD tested in the Condition I. In the forward-I task, the activity of the neuron increased when the stage moved to the window side (north) in Route 2 (*SI* = 4.2), but it did not increase in Route 1 (a). In the backward-I task, the activity of the neuron also increased during backward movement to the window side (north) in Route 2 (*SI* = 3.5), but it did not increase in Route 1 (b). Thus, the activity of this neuron increased during movement to the window side (north) regardless of the heading direction.

**Figure 7:**
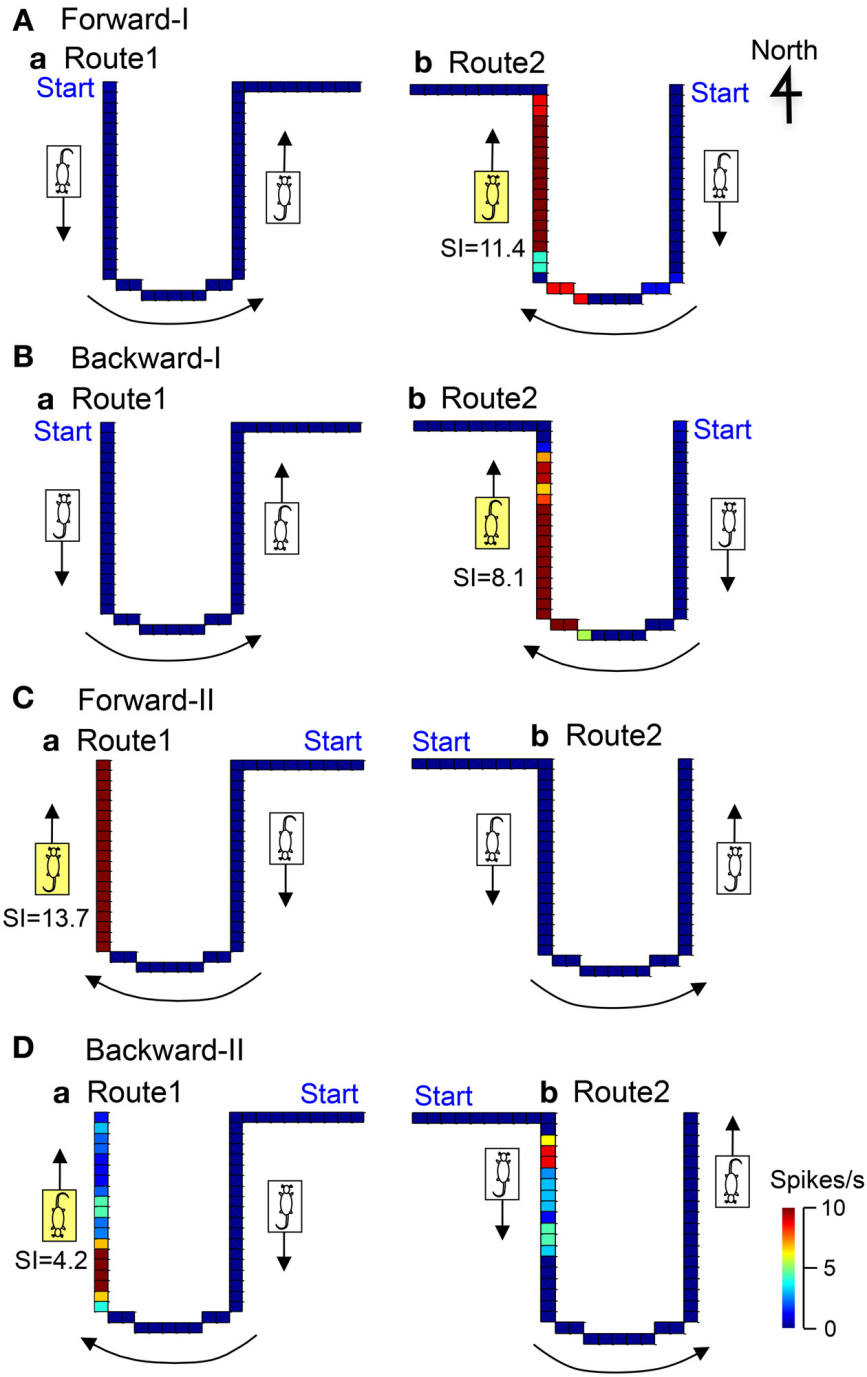
**Another example of a movement direction-related neuron in AD regardless of heading direction that was tested in both Condition I (A,B) and II (C,D).** In the Forward-I task **(A)**, the activity of the neuron increased when the stage moved to the window side (north) only in Route 2 but not when the stage move to the south in Routes 1 and 2. In the Backward-I task **(B)**, the activity of the neuron similarly increased when the stage moved to the window side (north) in Route 2. In the Condition II **(C,D)**, the activity of the neuron increased when the stage moved to the window side (north) in Route 1 but not when the stage moved to the door side (south) in Routes 1 and 2. Thus, the activity of the neuron increased when the stage moved to the window side (north) regardless of the facing direction of the rat. It was noted that the activity of the neuron increased when the stage moved just after clockwise-rotating movements (indicated by the curved arrows) but not after anticlockwise-rotating movement. The descriptions are the same as for Figure 4.

Figure 7 illustrates the representative data of another movement direction-related neuron in AD tested in both the Conditions I and II. In the Condition I, the activity of the neuron increased when the stage moved to the window side (north) in Route 2 in the forward-I (*SI* = 11.4) and backward-I (*SI* = 8.1) tasks. In the Condition II, the activity of the neuron increased when the stage moved to the window side (north) in Route 1 in the forward-II (*SI* = 13.7) and backward-II (*SI* = 4.2) tasks. Thus, the activity of this neuron increased during movement to the window side (north) regardless of the heading direction and the tasks.

### Effects of the routes on direction-related activity

Table 1 summarizes the results of route modulation. Of the 46 direction-related neurons (15 head direction-related, 6 forward movement-related, 7 backward movement-related, and 18 movement direction-related neurons), 22 direction-related neurons showed different movement-related responses depending on the routes (i.e., a significant interaction between direction and route). Of these 22 neurons, 18 showed maximal firing (i.e., preferred firing direction) in the common central stem. Of these 18 neurons, 11 showed significant modulation by the routes; the direction-related activity in the same place (i.e., common central stem) was different depending on the routes to and/or from the central stem (unpaired *t*-test, *p* < 0.05). The results in these 11 neurons indicate that route modulation was not ascribed to local factors in the area where preferred firing direction was observed, but attributed to differences in the routes (certain factors before and/or after the central stem).

The remaining 24 neurons showed similar direction-related responses in both Routes 1 and 2. Figure 4C shows an example of this type neurons in the LD tested in the Condition I. In the forward-I task, the activity of the neuron increased in the preferred direction when the rat faced the door side (south) in both Routes 1 and 2. The SI indexes in the preferred heading direction of the Routes 1 and 2 were 2.08 and 2.2, respectively. In the backward-I task, the activity of the neuron increased similarly when the rat faced the door side (south) in both routes. The SI indexes in the preferred heading direction of the Routes 1 and 2 were 1.6 and 1.4. The results indicate that this type neurons code heading direction regardless of the routes.

### Location of the direction-related neurons

**Figure 8:**
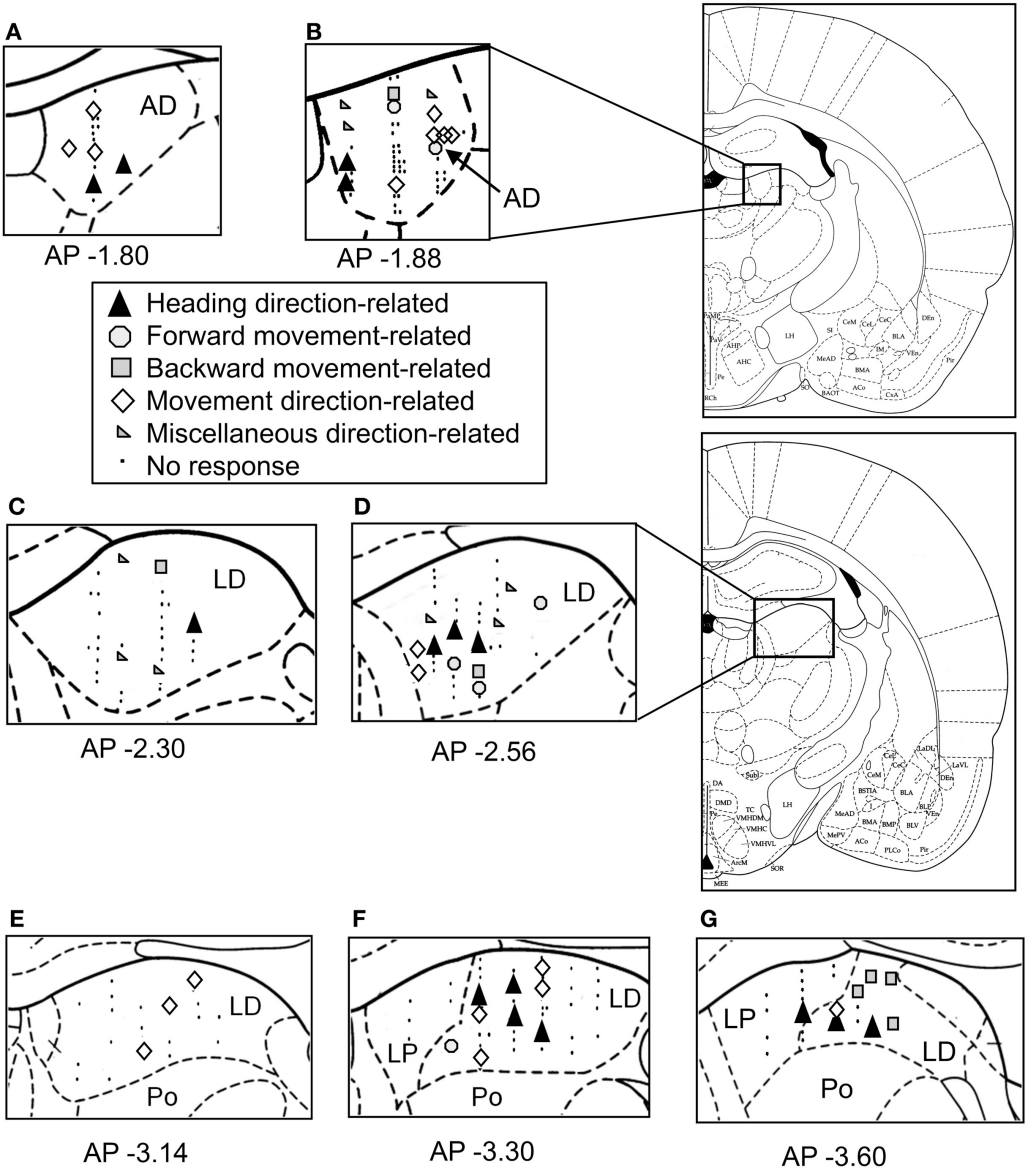
**Schematics of the locations of the recording sites and the categories of the direction-related neurons in the thalamus. (A–G)** The neurons are plotted on coronal sections of the right thalamus. The number below each section indicates the distance (mm) anterior from the interaural line. AD, anterodorsal thalamic nucleus; LD, laterodorsal thalamic nucleus; Po, posterior thalamic nuclei; LP, lateral posterior thalamic nucleus.

Figure 8 shows the histological localization of the direction-related neurons. All of these neurons were located in the AD and LD thalamic nuclei. The various types of direction-related neurons were intermingled and found in both the AD and LD thalamic nuclei. In the LD, there was a significant difference in recording positions along the anterior-posterior axis among the different types of direction-related neurons [One-Way ANOVA; *F*_(4, 32)_ = 3.765, *p* = 0.0128]. The *post-hoc* tests revealed that the miscellaneous direction-related neurons were located in the more anterior positions than the other types of the direction-related neurons except the forward movement-related neurons (Ryan's method, *p* < 0.05). In the AD, there was no significant difference in the recording positions among the different types of the direction-related neurons [One-Way ANOVA; *F*_(4, 13)_ = 0.832, *p* = 0.5283]. These results indicated that four types of the direction-related neurons (the facing direction-related, forward and backward movement-related, and movement direction-related neurons) were intermingled in both the AD and LD.

## Discussion

In the present study, we analyzed the effects of sensory conflict on the direction-related responses of the AD and LD thalamic neurons under conditions in which the motor/proprioceptive information indicated forward movements while the vestibular and visual information indicated backward movements. Of the 222 neurons, 55 facing and/or movement direction-related neurons were recorded from the AD and LD thalamus. These neurons showed complex spatial firing patterns in which the neuronal activity was dependent on the complex combinations of facing direction and movement direction.

### Heading direction-related responses

Of the 55 direction-related neurons, 15 (27.2%) neurons were heading direction-related neurons, and their activity was dependent on heading direction regardless of movement direction. Furthermore, the activity of more than half of the heading direction-related neurons (*n* = 10) was route-dependent. These characteristics of the heading direction-related neurons were different from those of HD cells in the AD and LD (Mizumori and Williams, 1993; Taube, 1995). HD cells in the AD and LD have been reported to discharge as a function of the rat's HD regardless of their behaviors and location in the environment (Mizumori and Williams, 1993; Taube, 1995). Vestibular information has been shown to play an important role in generating HD cell activity (Shinder and Taube, 2011), although previous studies have reported a powerful influence of visual cues (landmarks) on HD cell activity (Taube and Burton, 1995; Knierim et al., 1998). The response characteristics of these heading direction-related neurons suggested that neither visual (landmarks) nor ideothetic [visual (optic flow), vestibular, or proprioceptive] information can account for the responsiveness of this type of neuron. Because previous studies have reported that hippocampal lesions affect HD cell activity in the AD (Golob and Taube, 1999) and have suggested that the anterior thalamus might integrate hippocampal inputs (Aggleton et al., 2010), complex information, such as trajectory information, from the hippocampus may contribute to the complex responsiveness of this type of neuron.

However, the activity of the remaining five heading direction-related neurons increased regardless of the routes and regardless of the movement direction. The activity of this type of neuron might be strongly controlled by visual (landmarks) cues. This type of HD cell that is under predominately visual control has been reported previously (Goodridge and Taube, 1995; Dudchenko and Zinyuk, 2005).

### Forward and backward movement-related neurons

Six (10.9%) and seven (12.7%) neurons showed forward and backward movement-related responses, respectively. These neurons did show that optimal firing occurred only for certain combinations of heading-direction and movement in both forward and backward movement-related responses, respectively. That is, these responses were dependent on both heading and movement direction. These findings suggested that these neurons might encode both ideothetic information (vestibular information and optic flow; sensitivity to movement direction) and non-ideothetic information (landmarks; sensitivity to facing direction). These findings further suggested that forward movement-related neurons might correspond to HD cells that are under the control of both ideothetic and non-ideothetic cues (Taube, 2007).

In the backward tasks, the visual (optic flow) and vestibular information conflicted with motor/proprioceptive cues. In humans, when conflicts are introduced between vestibular and proprioceptive cues, spatial updating has been shown to be based on a weighted average of the two inputs (Frissen et al., 2011). According to this theory, the effects of visual (optic flow) and vestibular information might override the effects of motor/proprioceptive cues in this type of neuron. In addition, it is possible that backward movement-related neurons might encode complex patterns of conflicting sensory cues by learning due to repeated exposure to this task. Because these backward movement-related neurons showed sensitivity to both movement direction and facing direction, these neurons might also correspond to HD cells, although this type of neuron has not been reported previously. These cells might play an important role in heading in a conflicting condition.

### Movement direction-related neurons

Eighteen (32.7%) neurons showed movement direction-related responses that were dependent on movement direction regardless of heading direction. That is, the neurons fired when the stage moved in a particular direction, regardless of the heading direction of the rat in the forward and backward tasks. It was not clear what combination of sensory inputs contributed to these firing patterns; the neuron responded during forward translation in which visual (optic flow) and vestibular information matched the motor/proprioceptive information, while the same neuron also responded during backward translation in which visual (optic flow) and vestibular information conflicted with motor/proprioceptive information. Furthermore, the direction indicated by visual (optic flow) and vestibular information in the forward task was opposite to that in the backward task. One possibility was that these neurons might encode movement direction in an allocentric reference frame.

### Effects of the routes on the direction-related activity

Of the 46 direction-related neurons, 22 direction-related neurons showed different movement-related responses depending on the routes. Furthermore, of the 18 direction-related neurons with preferred firing direction in the common central stem, 11 showed significant modulation by the routes. The results indicate that certain factors before and/or after the central stem (factors due to route difference) affected direction-related activity in the central stem. These results provide a neurophysiological basis of anticipatory orienting responses, which were observed in not only forward but also backward locomotion in humans (Grasso et al., 1998).

It remains unknown what factors are involved in route modulation in most neurons with significant route modulation. However, this factor could be speculated in some movement direction-related neurons with route selectivity (e.g., Figure 7, Table 1). It is noted that the activity of these movement direction-related neurons increased in the different routes between the Conditions I and II (e.g., Figure 7) and that the directions of the rotation in the curved sections of the routes were opposite between the Conditions I and II. The activity of the neuron shown in Figure 7 increased after clockwise rotation regardless of the routes and tasks. These findings suggested that the activity of this type of neuron with route specificity might be dependent on movement trajectory. Thus, incoming instantaneous sensory inputs alone cannot account for all of complex responses of these neurons. That is, immediately preceding trajectory information might be required to form the complex responses in these neurons. Previous anatomical and behavioral studies suggest that the anterior thalamus and hippocampus are interdependent by multiple hippocampal-thalamic pathways (direct and indirect) and support episodic memory (Groen et al., 2002; Aggleton et al., 2010). Therefore, these neurons might receive trajectory information from the hippocampus.

### Diversity of the neuronal types

The present data indicated that there were various types of movement direction-related neurons, the activity of which was dependent on complex combinations of heading and movement directions. Four types of the direction-related neurons (the facing direction-related, forward and backward movement-related, and movement direction-related neurons) were intermingled, and located in both the AD and LD thalamic nuclei. Of the total 222 neurons 64 (28.8%) neurons were recorded from AD and 158 (71.2%) neurons were recorded from LD thalamus (e.g., Figure 8). There are no specific topographies within the AD and LD for the heading and/or movement-related neurons. A previous study reported that classic HD cells were localized in the dorsal LD thalamus, and the ventral LD receives inputs from the postsubiculum that processes information related to head direction, spatial location, and general movements (Mizumori and Williams, 1993). Complex responsiveness of the direction-related neurons in the present study might reflect these inputs.

Cho and Sharp (2001) have reported that space- and movement-related neurons show a heterogeneous mixture of neuronal types in the RSC; almost all of the neurons in the RSC are significantly correlated with spatial and movement-related variables (heterogeneous cell types). Based on these findings, they have suggested that this cortical region might play a role in path integration or navigational motor planning. Both the AD and LD thalamic nuclei are reciprocally connected with the RSC (Vogt and Miller, 1983; Sripanidkulcha and Wyss, 1986). Furthermore, lesion and electrical stimulation studies have suggested anterior thalamic involvement in contextual fear conditioning, spatial alternating, and temporal information processing (Charles et al., 2004; Hamani et al., 2010; Jankowski et al., 2013). All of these findings suggest that the AD and LD thalamic nuclei might be involved in not only heading functions but also other more complex spatial and mnemonic functions, and this is consistent with the diversity of the neuronal types reported in the present study.

### Methodological issues

The various response patterns of the direction-related neurons found in the present study might be ascribed to the artificial setup in the present study (head fixing and unnatural backward movement). However, it might be unlikely; first, the previous studies reported that although the backward movement increased hippocampal theta and sympathetic nervous activity, which corresponds to symptoms in motion sickness, the activity returned to the baseline level after repeated experience of this situation (Zou et al., 2009a; Aitake et al., 2011). This suggests that the rats could adapt to this situation. Second, another recent study has reported that AD HD cells of rats with their head painlessly fixed to a stereotaxic frame discharge normally during passive rotation, which was similar to those recorded in freely moving conditions (Shinder and Taube, 2011). Furthermore, other studies have reported that hippocampal place cell activities can be recorded from rats and mice with their heads fixed, as in the present study (Dayawansa et al., 2006; Harvey et al., 2009; Chen et al., 2013). These findings suggest that the fixation of the rat head itself might not affect AD neuronal activity. Third, a recent study reported that hippocampal place cell properties observed during backward movement are similar to those during forward movement (Cei et al., 2014), suggesting the rats could normally recognize a space even during backward movement.

In the present study, we tested the thalamic neurons with only limited head direction (south-north), while other researchers have tested thalamic neurons with all head directions (i.e., 360°) by rotating the stage upon which the rats were located (Zugaro et al., 2001b, 2002; Shinder and Taube, 2011). According to these previous studies, it is presumed that there are thalamic HD neurons that code directions other than south-north direction. The relatively fewer percentages of responsive neurons in the present study (10% of the thalamic neurons) might be ascribed to the usage of limited head directions in the present study. However, we found similar numbers of direction-dependent responses to the window and door sides. The rats moved in the fixed routes with the central stem that was 150 cm in length in the window-door (north-south) direction. Behavioral studies have suggested that animals learn to orient in relation to the overall shape of the testing environment or the principal axis of the environment, which passes through the centroid of a shape (i.e., the long axis) (Cheng, 1986; Cheng and Newcombe, 2005; Clark et al., 2012). The preferential responses to the one particular direction (door-window) (south-north direction) matched the principal axis of the route (the long axis of the routes) in the present study. With repeated exposure to this task, the anterior thalamic neurons might gradually develop direction-related responses, as has been reported by Dudchenko and Zinyuk (2005).

In the present study, rats were trained to run on the treadmill while the motion stage was translocated for 2–3 weeks, and each rat was usually tested every other day. After training, the rats were submitted to experimental sessions for 2 months. It is possible that the direction-related neuronal responses in the present study might reflect these learning since long-term potentiation and depression have been reported in the anterior thalamus (Aggleton et al., 2010). Further studies are required to test this idea.

## Conclusions

**Figure 9:**
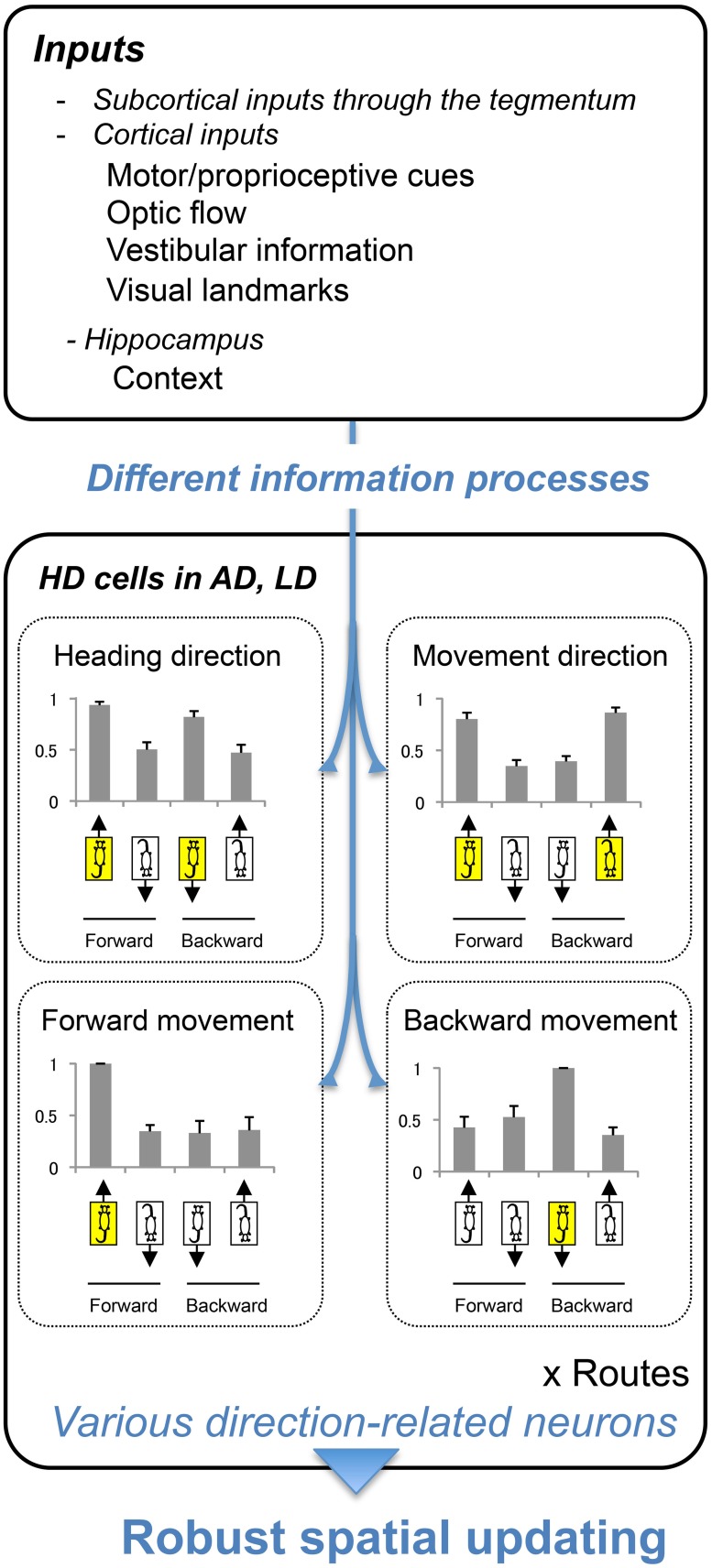
**A hypothesis of directional information processing in AD and LD.** AD and LD receive multisensory inputs (upper inset). By processing the information differently, different types of direction signals were generated (lower inset). These signals are used for robust spatial updating under different conditions, such as forward and backward translocations. Bar graphs indicate the normalized responses; responses in each directional situation (shown by the illustration of the rats indicating heading and movement directions) were divided by the maximal responses among the 4 directional situations. Error bars indicate s.e.m.

Figure 9 summarizes the present results. Histograms in Figure 9 indicate normalized response magnitudes to the directional stimuli. The present results indicated existence of four types of the direction-related neurons in the thalamus. Especially, two subpopulations of the HD cells in the AD and LD coded separately heading and movement directions (heading direction-related, and movement direction-related neurons) regardless of direction of translocation (forward or backward). These neurons might code directional information in an allocentric reference frame. Furthermore, the forward and backward movement-related neurons might code movement direction in an egocentric reference frame. These results suggest that the HD system can code different kinds of directional information separately when multiple sensory information contradicts. Previous studies suggest that the AD and LD receive multisensory inputs and these inputs are integrated to reduce the error of the head direction signal of the HD cells (Taube, 2007). The present study provides additional evidence with respect to a role of the HD system in directional processing; the rodent HD system extracts different types of directional information (Figure 9) in different reference frames in a conflicting situation. The previous studies suggest the rats could adapt to backward translocation (Zou et al., 2009a; Aitake et al., 2011), and hippocampal neurons showed plastic changes in place fields after repeated experience of backward translocation in the same setup as in the present study (Zou et al., 2009b). The HD neurons reported in the present study might play important roles for spatial updating during the backward translocation. Taken together, the present study suggests that the AD and LD generate complex direction-related signals for robust spatial updating under different conditions. Because the AD and LD nuclei serve as an important interactive for the limbic spatial learning system (Jankowski et al., 2013), the functions of the AD and LD nuclei might reflect those in other limbic areas. Future works and modeling will help to elucidate the complex function of the AD and LD thalamic nuclei.

### Conflict of interest statement

The authors declare that the research was conducted in the absence of any commercial or financial relationships that could be construed as a potential conflict of interest.
